# The Potential Role of Ecotoxicological Data in National Essential Medicine Lists: A Cross-Sectional Analysis

**DOI:** 10.3390/ijerph22040632

**Published:** 2025-04-17

**Authors:** Camila Heredia, Aine Workentin, Gillian Parker, Navindra Persaud

**Affiliations:** 1 MAP Centre for Urban Health Solutions, Li Ka Shing Knowledge Institute, St. Michael’s Hospital, Toronto, ON M5B 1T8, Canada; camila.heredia@unityhealth.to (C.H.); aine.workentin@unityhealth.to (A.W.); 2 CHSC Student Training Program, Institute of Health Policy, Management and Evaluation, Dalla Lana School of Public Health, University of Toronto, Toronto, ON M5T 3M6, Canada; gillian.parker@utoronto.ca; 3 Department of Family and Community Medicine, Faculty of Medicine, University of Toronto, Toronto, ON M5B 1X2, Canada

**Keywords:** ecotoxicity, essential medicine lists, bioaccumulation, persistence, essential medicines, environment

## Abstract

Background: Medicines affect the environment throughout their lifecycle, from production and distribution to use and disposal. They contribute to the pollution of air, water, and soil, impacting ecosystems and human health. Recognizing these risks, regulatory bodies and organizations have highlighted pharmaceutical pollution as a global concern, emphasizing the need for environmental risk assessments and sustainable practices. Methods: This study reviewed the essential medicines lists (EMLs) from 158 countries and examined the available ecotoxicological data. Medicines with high bioaccumulation, persistence, and toxicity were identified and cross-referenced with their inclusion in EMLs. Additionally, we analyzed the presence of alternative medicines with similar therapeutic effects but potentially lower environmental risks. Results: Five medicines—ciprofloxacin, ethinylestradiol, levonorgestrel, ibuprofen, and sertraline—were selected as illustrative examples due to their high environmental persistence and toxicity. All were listed in the 2023 WHO model list, with ciprofloxacin appearing in 94.3% of national EMLs. Conclusions: This study underscores the limited availability of ecotoxicological data, which hinders environmental risk assessment for medicines. EMLs could serve as a tool to enhance the awareness and data mobilization of pharmaceutical pollution. Incorporating environmental criteria into EMLs could support more sustainable medicine selection and regulatory practices.

## 1. Introduction

Medicines affect the environment during production, packaging, distribution, use, and after excretion [1,2,3,4,5]. They can directly or indirectly pollute air, water, and soil, which can harm plants, animals, and people who do not directly consume the medicines [6]. The harmful environmental effects of medicines are one of the numerous contributions of the healthcare sector to ecosystem pollution and climate change [7]. There is growing recognition of the importance of considering the environmental impact of healthcare when designing services and selecting products, including medicines [8]. In 2020, the United Nations’ Assessment Report on Issues of Concern proposed broadening its focus to include pharmaceuticals in the environment, highlighting pharmaceutical pollutants and antimicrobial resistance [9]. That same year, the World Health Organization (WHO) introduced a document outlining considerations for addressing the environmental impact of pharmaceutical manufacturing, particularly targeting waste and wastewater management to prevent antimicrobial resistance [10]. In addition, market authorization agencies, such as the European Medicines Agency (EMA) and organizations like the Organization for Economic Cooperation and Development (OECD) have adopted Environmental Risk Assessment (ERA) requirements for pharmaceutical products. The OECD’s 2019 report on pharmaceutical residues in freshwater stresses the need for deeper understanding of the environmental impacts of medicines and calls for stronger international collaboration, clearer accountability, and the development of policies to prevent and address emerging challenges [11]. Addressing pharmaceutical pollution will require action throughout the product lifecycle, with upstream stakeholders such as producers and regulators emphasizing transparency, robust data, and sustainable practices, while downstream actors like prescribers work to mitigate overuse and improper prescribing as part of a broader solution [5]. While it is clear that medicines can have harmful effects on the environment, and regulators and health organizations have recognized these issues, there is currently limited and inconsistent information available about the ecotoxicological impacts of medicines [12].

Essential medicines lists (EMLs) are developed to be a resource to support countries to meet the priority health needs of their populations. The WHO maintains a model list of essential medicines, and more than 150 countries have developed lists that guide medicine availability and accessibility for billions of people globally. Today, EMLs are developed primarily based on clinical considerations and practical issues such as population need, efficacy, safety, effectiveness, quality, costs, and availability. These lists are typically updated annually, and medicines are removed or deselected if better alternatives become available or if a medicine is deemed to be ineffective or harmful [13].

We conducted a review of the selected medicines listed in EMLs and analyzed the reported ecotoxicological data. Our goal was to identify medicines with significant environmental impacts. We then examined medicines with similar clinical effects to assess whether less environmentally harmful alternatives could be recommended. This approach could help integrate ecotoxicological criteria into EMLs for more sustainable medicine selection.

## 2. Materials and Methods

### 2.1. Data Sources

#### 2.1.1. Essential Medicines

Essential medicines were identified through the database we developed (essentialmeds.org) that contains 158 essential medicines lists, identifying prioritized medications for five billion people [14,15]. In May 2023, we searched online for national essential medicines lists (NEMLs), government websites, and contacted healthcare officials and experts to collect medicine lists from countries where WHO operates. We included both outpatient and inpatient lists across all levels of care and in all languages, excluding documents that were only prescribing guidelines.

We extracted medicines using International Nonproprietary Names (INNs), translating non-English names and standardizing entries by ignoring salt forms. Unlike the previous study, which was fully manual, this update used a combination of automated and manual methods. A web scraper matched medicine names with Anatomical Therapeutic Chemical (ATC) codes through a multi-step search strategy, incorporating proxies and randomized searches to enhance reliability.

All extracted data underwent manual review by two researchers. To verify accuracy, 400 randomly selected data points were checked, yielding an error rate of 0.75%. We did not collect details on medicine usage, doses, or formulations but documented general list information, such as publication dates and objectives. Diagnostic agents, antiseptics, disinfectants, saline solutions, and naturopathic medicines were excluded.

#### 2.1.2. Health Expenditures

Health expenditure data were obtained from the Global Health Observatory, except for Somalia and the Democratic People’s Republic of Korea, where information was not reported by WHO, so the data was extracted from the Amnesty International Press Release and a nonprofit media organization [16,17,18]. Most of the data pertained to the year 2023; if 2023 records were unavailable, information from the nearest available year to 2023 was accessed.

#### 2.1.3. Ecotoxicological Data on Medicines

Ecotoxicity data on medicines were identified through scholarly and gray literature, jurisdictional lists (e.g., European Union’s published watch list, Stockholm County Council’s Environmental Program 2017–2021) [19], and environmental data websites (e.g., Janusinfo.se, Fass.se) [20,21]. Sources were selected based on their relevance, credibility, and accessibility, ensuring the inclusion of publicly available regulatory and research-based data while excluding proprietary or unpublished information.

### 2.2. Data Analysis

We identified medicines reported to have the highest values in the parameters of bioaccumulation, persistence, and toxicity (high bioaccumulation, environmentally persistent, very high toxicity) [22]. Bioaccumulation refers to a substance’s tendency to build up in the fatty tissues of aquatic organisms. It is evaluated using the partition coefficient (n-octanol/water), commonly expressed as log Kow (or log Pow). A substance with a log Kow of 4.5 or higher is considered to have a strong potential for bioaccumulation, as determined by OECD tests 107 or 117.

Persistence describes a substance’s resistance to breaking down in aquatic environments. It is measured using degradability tests, such as OECD test guidelines 301 and 308, or other equivalent methods.

Toxicity refers to a substance’s potential to harm aquatic life. It is assessed through toxicity tests conducted on three key trophic levels in the food chain—algae, crustaceans, and fish—using OECD acute toxicity test guidelines 201, 202, and 203, or similar standards. For chronic toxicity, tests such as OECD guidelines 201, 210, and 211 are used, with the most sensitive species data being considered in the evaluation [22].

These medicines were cross-referenced with medicines included in national essential medicines lists.

## 3. Results

From our review of the essential medicines lists and available sources of ecotoxicological information, we selected 36 potentially harmful medicines: acetaminophen, acetylsalicilic acid, amoxicillin, azithromycin, ciprofloxacin, citalopram, clarithromycin, clindamycin, clotrimazole, diazepam, diclofenac, erythromycin, estradiol, ethinyilestradiol, felodipine, fluconazole, fluoxetine, flupentixol, glibenclamide, haloperidol, hydroquinone, ibuprofen, irbesartan, levonorgestrel, meclozine, metformin, miconazole, nitrous oxide, oxazepam, risperidone, roxithromysin, sertraline, sulfamethoxazole, tetracycline, trimethoprim, and venlafaxin. We then chose five medicines as illustrative examples of medicines that appear on EMLs and have a high reported bioaccumulation, environmentally persistence, or very high toxicity. The five example medicines fall into four categories: fluoroquinolones (e.g., ciprofloxacin), sex hormones (e.g., ethinylestradiol and levonorgestrel), propionic acid derivative anti-inflammatories (e.g., ibuprofen), and selective serotonin reuptake inhibitor antidepressants (e.g., sertraline).

### 3.1. Reported Ecotoxicological Risks of the Example Medicines

Ibuprofen. Propionic acid derivatives are a class of nonselective, nonsteroidal anti-inflammatory medicines, such as ibuprofen, ketoprofen, and naproxen, with analgesic and antipyretic effects. Enzymes in human and animal bodies do not completely metabolize the medicine, so its elimination includes ibuprofen and its metabolites (which are also toxic, such as carboxyibuprofen, hydroxyibuprofen, and carboxyhydratropic acid) [23]. Ibuprofen, one of the most commonly prescribed medicines in the world, is found in very high concentrations and exhibits significant toxicity in samples taken from wastewater treatment plants and bodies of water across the globe and currently cannot be removed using conventional water treatment methods [21]. Acute and long-term effects include changes in growth rate, behavior and reproduction modifications, and biochemical alterations across various aquatic organisms [23]. Ibuprofen detection and removal are key measures to reduce its environmental damage, including chemical (ozonation, gamma radiolysis, advanced oxidation processes, degradation), physical (adsorption using activated carbon), and biological methods (biodegradation) [23,24].

Other anti-inflammatory medicines (apart from the propionic acid derivatives naproxen and ketoprofen) include diclofenac, celecoxib, etoricoxib, and meloxicam. Ketoprofen, although described as posing a low environmental risk, has high chronic toxicity and potentially persists in the environment [20]. Diclofenac degrades slowly in the environment and also poses a high environmental risk [20]. Celecoxib, etoricoxib, and meloxicam may be environmentally harmful and some sources indicate coxibs have similar risks as ibuprofen [20,25]. Some bacterial strains with enzymes that can break down naproxen have been discovered, and therefore, currently, using naproxen has been reported as posing a lower environmental risk [20,26].

Ciprofloxacin. Fluoroquinolones are wide spectrum antibiotics, highly prescribed in hospitals and ambulatory settings. They are excreted largely unchanged, up to 70%, and when introduced into the environment, they can encourage the development of resistance in microbial populations [27]. Ciprofloxacin is a frequently utilized fluoroquinolone to treat bacterial infections such as urinary tract infections and pneumonia. Ciprofloxacin persists in water and is resistant to biodegradation, raising concerns for public health and ecological stability [28]. High concentrations of ciprofloxacin in the environment can result in genotoxic effects on aquatic organisms. This raises concerns about long-term ecological effects and the possible transfer of antibiotic resistance genes to human pathogens via environmental reservoirs [28]. Although ciprofloxacin concentration in soils was not found to be high, it inhibits active and growing microorganisms and retains its biological activity over time [29]. Ciprofloxacin may promote the development and spread of resistance in bacterial pathogens [29].

A proposed solution includes selecting antibiotics with a narrow spectrum that are effective for the intended treatment, such as nitrofurantoin (considered to pose a lower environmental risk) [20]. Although other fluoroquinolones such as levofloxacin, norfloxacin, and ofloxacin can be suggested as therapeutic alternatives with a similar clinical effect, they also report environmental risks [20].

Ethinylestradiol and levonorgestrel. Sex hormones, such as estrogens and progestins, are chemical substances that regulate sexual development, reproduction, and other functions related to sexual characteristics. Ethinylestradiol is a synthetic estrogen while levonorgestrel is a synthetic progestin, both widely used in contraceptive pills. Estrogens present in the environment can have harmful effects on organisms, including animals, by causing issues such as feminization, the disruption of natural reproductive processes, reduced overall health, imbalances in pro-apoptotic and anti-apoptotic regulation, and even the development of cancer, significantly impacting animal well-being [30]. Similarly, progestins are also regarded as emerging micropollutants (newly recognized contaminants found in the environment, often at very low concentrations, but with potential risks to ecosystems and human health) in aquatic ecosystems, where they are typically found at concentrations in the nanograms per liter (ng/L) range [31]. Available data indicate that synthetic hormones are more persistent in the environment than natural ones and may therefore pose a greater environmental concern. They enter the environment from various sources and have been shown to significantly impact the reproductive health of various aquatic vertebrates and plants [32]. Synthetic estrogens are present at polluting levels at sites near wastewater treatment facilities and in groundwater at various locations globally (their widespread distribution in the environment occurs due to their incomplete removal in sewage treatment plants and leaching, reaching surface and groundwater through the release of domestic sewage into waterways, and effluents from pharmaceutical industries) [32]. Consequences include the activation of androgen receptors in fish, leading to the development of male secondary sex characteristics in females of other species [33].

Estradiol and estriol are also estrogens, with the former having a described lower risk of environmental impact, and the latter with no formal damage quantification [20,34]. Similarly, some progestogen alternatives to levonorgestrel include desogestrel (moderate environmental risk) [20], etonogestrel (moderate environmental risk) [20], medroxyprogesterone (androgenic damage to aquatic organisms) [35], and norethisterone (moderate to high environmental risk) [20]. Therefore, it is currently challenging to identify clinical alternatives to synthetic estrogens and progestogens that have little to no environmental impact.

Sertraline. Selective serotonin reuptake inhibitors are widely used antidepressants, such as sertraline, citalopram, fluoxetine, paroxetine, and others. They are present in various environmental compartments, such as wastewater, surface water, groundwater, drinking water, and sediments, highlighting the increasing concern surrounding these emerging environmental pollutants [36]. Sertraline, used to treat panic disorder and depression, has been found in fish at concentrations similar to therapeutic levels in humans [37]. It has been described to have negative effects on animal behavior and reproduction, such as reducing and delaying fish locomotion and learning, respectively [37]. Sertraline presence has been reported not only in surface water samples but also in several fish species, as it directly reaches water or undergoes a metabolic transformation that ends in metabolites with similar effects [37]. The environmental impacts of sertraline are likely to increase, as serotonin reuptake inhibitors’ global consumption is also increasing [38].

Bupropion, citalopram, duloxetine, escitalopram, fluoxetine, mirtazapine, paroxetine, and venlafaxine are other antidepressants with high ecotoxicity and some bioaccumulation potential, making them a clinical alternative to sertraline but also presenting environmental risks [20].

### 3.2. Inclusion of Example Medicines in Essential Medicine Lists

All of the five example medicines were listed in the 2023 WHO model list [39]. Ciprofloxacin was the medicine listed by most of the countries (149 EMLs; 94.3%), while sertraline was the least-cited medicine (70 EMLs, 44.3%). Among the total 158 countries, 55 listed all five example medicines (34.8%), while only Japan and Spain (2; 1.3%) cited none of them (median: 4, IQR: 4–5) (Table 1). When searching the database for alternatives to the five example medicines based on the ATC codes, we found that Japan listed norfloxacin and ofloxacin as alternatives to ciprofloxacin and conjugated estrogens as an alternative to ethinylestradiol. However, no alternatives were found for ibuprofen, levonorgestrel, or sertraline. In contrast, Spain did not list any alternatives for any of the five medicines.

Out of the 147 countries listing ibuprofen, 36.1% (53) also included naproxen, 20.4% (30) also listed celecoxib, only 7.5% (11) included etoricoxib, 19.7% (29) mentioned meloxicam, 32% of the countries (47) also included ketoprofen, while most (83.7%, 123) also listed diclofenac in their EMLs.

Out of the 149 countries listing ciprofloxacin, 78.5% (117) also included nitrofurantoin, 75.2% (112) included levofloxacin, 28.9% (43) included norfloxacin, and more than half (58.4%, 87) included ofloxacin in their EMLs.

Of the 137 countries listing ethinylestradiol, 48.9% (67) also included estradiol, while only 12.5% (17) included estriol.

Among the 135 countries listing levonorgestrel, only 21.5% (29) also included desogestrel in their EMLs, 35.6% (48) mentioned etonogestrel, while most (93.4%, 126 countries) included medroxyprogesterone and (80.7%, 109) norethisterone.

Among the 70 countries listing sertraline, 31.4% (22) also included bupropion, 37.1% (26) included citalopram, 38.6% (27) included duloxetine and mirtazapine, half of the 70 countries (50%, 35) also mentioned escitalopram, similar with paroxetine, and the majority (94.3%, 66) included fluoxetine, while 54.3% (38) also included venlafaxine.

We also analyzed countries listing alternatives for ibuprofen. Nine countries listed celecoxib, etoricoxib, meloxicam, and naproxen (Czechia, Estonia, Greece, Ireland, Kazakhstan, Maldives, Mexico, Oman, and Slovenia), while the WHO 2023 model list and 87 countries listed none of them.

### 3.3. Healthcare Expenditure for Countries That List the Example Medicines

**Figure 1:**
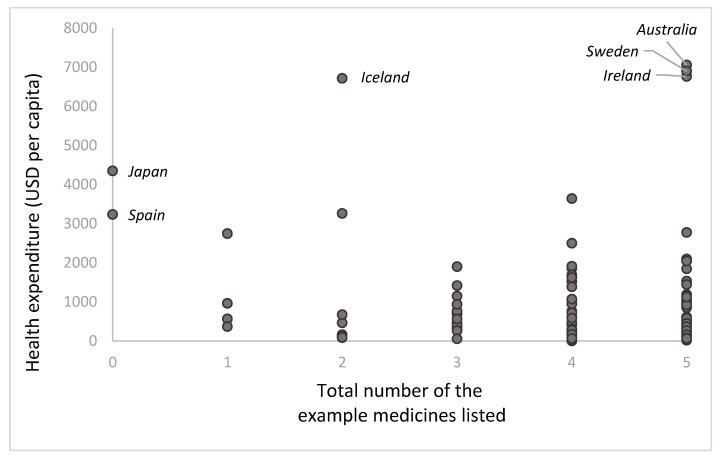
Health expenditure (USD per capita) by total number of the five example medicines listed.

**Figure 2:**
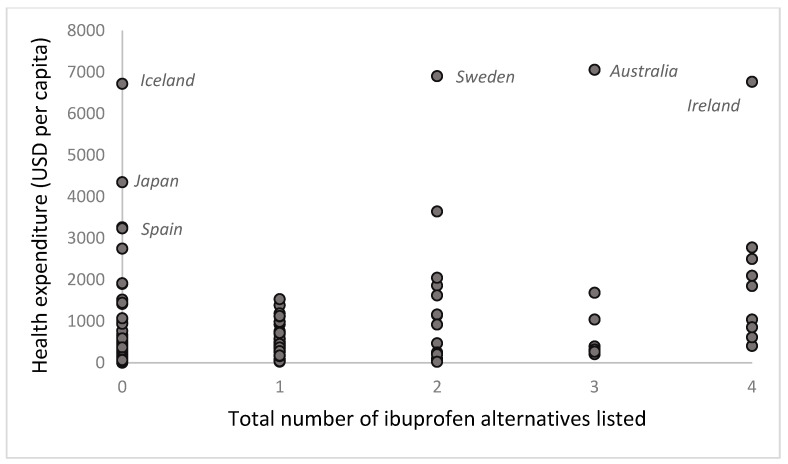
Health expenditure (USD per capita) by total number of ibuprofen alternatives (celecoxib, etoricoxib, meloxicam, and naproxen) listed.

From the 57 countries listing all or none of the five example medicines (Table 2), most have low health expenditure. Japan and Spain (which did not list any of the five example medicines) have much higher per capita health expenditure [16,40,41]. When analyzing per capita health expenditure by the number of the example medicines listed (Figure 1), we find that health expenditure per capita does not have a straightforward relationship with the number of example medicines listed. Countries with higher health expenditure per capita are not necessarily the ones listing more medicines (Japan and Spain list none of them, while Australia, Sweden, and Ireland list all of them). Most countries list four or five of the example medicines and have low health expenditure per capita. To illustrate, there is no direct relationship between per capita health expenditure and the number of alternatives to ibuprofen. Some countries with high health expenditure list none of the alternatives (Iceland, Japan, and Spain), while Sweden lists only celecoxib and naproxen; Australia lists all except etoricoxib, and Ireland lists all of them (Figure 2).

## 4. Discussion

Through this analysis, we reviewed the reported ecotoxicological data for five example medicines listed on EMLs. Our goal was to explore the feasibility of identifying medicines on EMLs with environmental harms and identifying clinical alternatives or criteria for recommending less environmentally harmful medicines. The results of this review demonstrate the limitations of the available ecotoxicological data. To strengthen the practical relevance of this study, exploring concrete strategies or real-world case studies on policy integration would also be valuable. Highlighting examples where countries or regulatory agencies have effectively integrated environmental considerations into EMLs could offer meaningful lessons and best practices. Additionally, examining existing policy frameworks that facilitate the inclusion of ecotoxicological data in pharmaceutical regulation would help illustrate viable pathways for broader adoption. While the proposal to incorporate environmental impact data into EMLs is promising, further discussion on potential barriers—such as challenges in data harmonization, regulatory alignment, and stakeholder buy-in—would provide a more comprehensive perspective on the feasibility and implementation of this approach.

The five example medicines represent commonly prescribed, used, and listed medicines from four critical classes. All five example medicines were listed in the 2023 WHO model list with one third of the included countries listing all five. In addition, our analysis of per capita health expenditures highlighted that these expenditures do not have a straightforward relationship with the number of example medicines listed.

### 4.1. Availability and Quality of Ecotoxicological Data

Our review demonstrated that environmental data are scant for medicines, and rigorously produced and validated data for a group of medicines in a class are not currently available. As demonstrated by our review, currently available published studies often contain many variables, including studies conducted in different jurisdictions, evaluating different doses or administrations, or using different methodologies. In addition, the sources we surveyed provided data from varied origins; often, reported ecotoxicological data were a direct republication of manufacturer-provided information. This lack of available and validated environmental data for medicines has been recognized in the literature [5,42] and impacts medicine regulation across the lifecycle, including at market authorization [43,44] and health technology assessment [45,46,47]. This lack of data currently hinders comparative analysis, but this information can be used to increase awareness of the environmental harms from medicines. While determining environmentally preferable medicines or developing environmental criteria to list/delist essential medicines is not possible at this time, much can be done to leverage EMLs to support awareness and encourage data production and mobilization to reduce the environmental harms from medicines.

### 4.2. Opportunities for EMLs to Support Efforts to Reduce the Environmental Harms from Medicines

Health technology assessments (a similar tool to EMLs and often used in high-income countries in lieu of EMLs) are also dealing with the challenge of a lack of data and lack of methods to assess and use the environmental data on medicines. One approach, increasingly leveraged to include the environmental impacts of a health technology, such as medicines or medical devices, is the “information conduit” approach [45]. It involves an agency republishing environmental data in a report that is in the public domain or has been submitted to the agency (e.g., by a manufacturer), without further assessment of the data [45]. It is argued that in addition to increasing awareness, this approach may also “*facilitate more environmentally informed decision-making at other levels of the health system (e.g., by companies, clinicians, payers, or patients)*” [45].

EMLs could act as information conduits for the ecotoxicological information of medicines, thereby encouraging manufacturers and regulators to produce and share these data. The WHO may be able to obtain the ecotoxicological data submitted by manufacturers at market registration, as the WHO currently requires evidence of market registration for inclusion in its model list [13]. This approach is similar to the Janus Info database, which collects and publishes this type of data for use in Sweden (this database can also be accessed and used by anyone).

This approach also aligns with the Wise List used in Stockholm County, Sweden, to share and mobilize ecotoxicological data for medicines. The Wise List is based on the principle that “*a pharmaceutical substance with a small or moderate impact on the environment should be recommended before a substance lacking in environmental information in order to promote manufacturers who provide environmental information*” [48]. Thus, incorporating information about ecotoxicological effects in EML listings could eventually lead to more complete environmental impact information being available. This approach could also have additional benefits as it has been argued that publishing environmental information may encourage manufacturers to develop “greener”, less environmentally harmful medicines [49], and as EMLs are used to inform the development of clinical practice guidelines (CPGs) and drug formularies, once validated ecotoxicological data on the majority of medicines in a class have been compiled, they can be leveraged by CPGs and formularies [13].

#### Strengths and Limitations

This analysis reviewed the available ecotoxicological information for a sample of five highly prescribed and used medicines belonging to important medicine classes, widely used by both adult and pediatric populations. Currently the available information about the environmental impacts of medicines is limited and much of the information about environmental effects comes from drug manufacturers and other industry sources. As a cross-sectional analysis, we were unable to assess how medicine listing changed over time. We did not assess whether medicines are prescribed appropriately, and this may be particularly important for antibiotics such as ciprofloxacin.

## 5. Conclusions

Through this review we explored the possibility of leveraging EMLs to address environmental harms from medicines. Our analysis revealed that the available data are currently inadequate to recommend environmentally preferable medicines or include environmental impacts as list criteria for EMLs. In light of these results, we explored the possibility that EMLs can be leveraged as an information conduit to disseminate information on the environmental harms from medicines. The inclusion of environmental impact data for medicines in EMLs can encourage manufacturers and regulators to produce rigorous and transparent data to support efforts to reduce the environmental harms from medicines. Future studies may provide further insights into the different approaches taken by different countries, including, for example, why Spain and Japan listed none of the five example medicines reported to be environmentally harmful. Strategies such as cross-referencing multiple databases, applying predictive modeling, or conducting systematic analyses of existing research could help detect discrepancies and enhance data reliability. Finally, working with regulatory bodies and independent research organizations could offer additional verification, contributing to a more thorough and trustworthy evaluation of pharmaceutical ecotoxicity.

## Figures and Tables

**Table 1:** Characteristics of example medicines.

	No. of Countries Listing It	Persistence (According to OECD’s Test Guidelines (Test 301, 308) or Corresponding Other Degradability Tests) [22]	Bioaccumulation [22]	Toxicity [22]	No. of Alternatives (Similar ATC Code)	Clinical Alternatives
Ciprofloxacin	149	Potentially persistent	Low	Very high chronic	25	Levofloxacin, nitrofurantoin, norfloxacin, ofloxacin
Ethinylestradiol	137	Degrades in the environment (the half-life of ethinylestradiol ranges from 4.0 to 5.9 days in water and from 24 to 36 days in sediment or the entire system)	High	Very high chronic	8	Estradiol, estriol
Ibuprofen	147	Degrades in the environment (meets the ready biodegradation test requirements, though there is some uncertainty regarding the 10-day window criterion)	Low	High chronic	23	Celecoxib, diclofenac, etoricoxib, ketoprofen, meloxicam, naproxen
Levonorgestrel	135	Persistent	Below high limit	Very high chronic	10	Desogestrel, etonogestrel, medroxyprogesterone, norethisterone
Sertraline	70	Degrades in the environment (after 45 days of biodegradation using the activated sludge method, 9–32% of sertraline persists)	No potential	Very high acute	9	Bupropion, citalopram, duloxetine, escitalopram, fluoxetine, mirtazapine, paroxetine, venlafaxine

**Table 2:** Country characteristics.

	Total of Five Example Medicines	Total No. of Medicines on List	Health Expenditure USD per Capita (2021)
Algeria 2023	5	516	205
Antigua and Barbuda 2022	5	334	923
Australia 2023	5	787	7055
Colombia 2019	5	594	558
Cuba 2018	5	469	1186
Dominica 2022	5	334	482
Dominican Republic 2018	5	386	417
Ecuador 2019	5	424	494
El Salvador 2020	5	272	442
Estonia 2012	5	405	2095
Eswatini 2012	5	312	280
Ethiopia 2020	5	440	26
Fiji 2015	5	291	250
Ghana 2017	5	400	100
Greece 2007	5	918	1846
Grenada 2022	5	334	505
Guinea-Bissau 2020	5	421	69
Honduras 2018	5	351	254
Iran (Islamic Republic of) 2017	5	955	393
Ireland 2023	5	740	6764
Jamaica 2015	5	445	372
Lebanon 2018	5	341	307
Libya 2019	5	538	381
Madagascar 2019	5	414	18
Malaysia 2023	5	428	487
Maldives 2021	5	853	1039
Mauritania 2021	5	326	89
Mexico 2017	5	794	611
Mongolia 2020	5	439	316
Montenegro 2020	5	535	985
Morocco 2017	5	395	221
Nauru 2010	5	230	1530
Oman 2020	5	793	853
Pakistan 2021	5	504	43
Palau 2017	5	278	2045
Peru 2018	5	451	412
Philippines 2022	5	528	203
Poland 2017	5	497	1159
Republic of Moldova 2021	5	506	410
Rwanda 2022	5	393	60
Saint Kitts and Nevis 2022	5	334	1114
Saint Lucia 2022	5	334	585
Saint Vincent and Grenadines 2022	5	334	448
Saudi Arabia 2020	5	525	1442
Serbia 2022	5	697	919
Slovenia 2017 + 2023	5	931	2775
Sri Lanka 2019	5	188	166
Sudan 2014	5	508	22
Sweden 2023	5	309	6901
Thailand 2021	5	583	364
Trinidad & Tobago 2019	5	467	1125
Tunisia 2012	5	642	265
Uzbekistan 2021	5	402	157
Zambia 2020	5	338	75
Zimbabwe 2020	5	301	63
Japan 2018	0	122	4347
Spain 2019	0	39	3234

## Data Availability

The raw data supporting the conclusions of this article will be made available by the authors on request.

## Abbreviations

The following abbreviations are used in this manuscript: CPG: Clinical Practice Guidelines; EMA: European Medicines Agency; EMLs: Essential Medicines Lists; ERA: Environmental Risk Assessment; OECD: Organization for Economic Cooperation and Development; WHO: World Health Organization.
